# Plasma Prolidase Activity and Oxidative Stress in Patients with Parkinson's Disease

**DOI:** 10.1155/2015/598028

**Published:** 2015-08-09

**Authors:** Akhilesh Kumar Verma, Janak Raj, Vivek Sharma, Tej Bali Singh, Shalabh Srivastava, Ragini Srivastava

**Affiliations:** ^1^Department of Biochemistry, Institute of Medical Sciences, Banaras Hindu University, Varanasi, Uttar Pradesh 221005, India; ^2^Department of Neurosurgery, Institute of Medical Sciences, Banaras Hindu University, Varanasi, Uttar Pradesh 221005, India; ^3^Division of Biostatistics, Department of Community Medicine, Institute of Medical Sciences, Banaras Hindu University, Varanasi, Uttar Pradesh 221005, India; ^4^Department of Oral Pathology, Jaipur Dental College, Jaipur, Rajasthan 302020, India

## Abstract

Prolidase deficiency has been related to mental retardation and oxidative stress. The study aimed to observe plasma prolidase activity (PPA), total oxidant status (TOS), total antioxidant status (TAS), and oxidative stress index (OSI) in patients with Parkinson's disease (PD). 240 subjects with PD and 150 healthy volunteers were considered as cases and controls, respectively. PPA, TOS, TAS, and OSI were measured spectrophotometrically. PPA and TAS in cases were more significantly decreased than controls (*P* < 0.01), while TOS and OSI were significantly increased (*P* < 0.001). In cases, nonsignificant, positive correlation was observed between PPA and TOS and OSI while significant, negative correlation was observed between PPA and TAS (*P* = 0.047). PPA in cases was nonsignificantly decreased with increased duration of PD (*P* = 0.747) while TAS was significantly decreased (*P* < 0.001) and TOS and OSI were significantly increased (*P* < 0.001). It was observed that higher age groups had decreased PPA, and TAS and increased TOS and OSI compared to lower age groups in cases. In summary, patients with PD have decreased PPA and increased oxidative stress compared to healthy volunteers. PPA was associated with oxidative stress markers in patients with PD. Decreased PPA and TAS and increased TOS and OSI were associated with progression of disease and higher age.

## 1. Introduction

Parkinson's disease (PD) is the second most common neurodegenerative disorder that mostly affects old people [1]. Some decades ago, the pathogenesis of PD was not clear. But with time, evidences like oxidative damage, inflammation, and nitrosative stress are found to be contributing factors [1–3]. Increased oxidative stress and inflammatory mediators such as reactive oxygen species, reactive nitrogen species, proinflammatory cytokines, and complement component have been reported in substantia nigra as well as in cerebrospinal fluid of patients with PD [3]. Thus, proinflammatory response can lead to the inflammation mediated degeneration of neurons in patients with PD [3, 4].

A cytosolic exopeptidase, prolidase, cleaves iminopeptides at carboxy-terminal proline or hydroxy-proline and is actively involved in collagen metabolism [5]. It has been reported that prolidase activity and inflammation can be correlated to each other during the process of fibrosis [6]. Prolidase activity has been also associated with oxidative stress in different diseases [7–11]. A report of Mantle et al. suggested that proline endopeptidase activity decreases in brain tissue of patients with Alzheimer's disease, Parkinson's disease, and Lewy body dementia compared to healthy individuals [12]. In the case of Alzheimer's disease, brain tissue proline endopeptidase activity decreased [12], while serum prolidase (proline exopeptidase) activity was increased with increase in oxidative stress [7]. It is well accepted that, as Alzheimer's disease, oxidative stress is found to be increased in the patients with Parkinson's disease (PD) [7, 13]. There are no previous studies present in literature regarding prolidase activity in the patients with PD. Thus for evaluating plasma proline exopeptidase activity in patients with PD, we aimed to investigate the correlation between plasma prolidase activity and oxidative stress markers (including total oxidant status, total antioxidant status, and oxidative stress index) in patients with PD.

## 2. Materials and Methods

The study was carried out in the Department of Biochemistry, Institute of Medical Sciences, Banaras Hindu University, Varanasi, India. The cases were provided by the department of neurosurgery. The study was approved by our institutional ethical committee and informed consent was taken from every studied subject.

### 2.1. Patients Selection

A total 390 subjects of cases and controls of matched age and sex were selected. Out of 390 subjects, 240 patients were cases of Parkinson's disease (180 males and 60 females) who were enrolled in Outpatients Department (OPD) of Neurosurgery, Institute of Medical Sciences, Banaras Hindu University, Varanasi, India. The age group was between 35 and 70 years with mean of 56.39 ± 8.92 years. The diagnosis of Parkinson's disease was made by our expert clinician. The range of duration of disease for PD was from 6 months to 14 years. 150 healthy subjects (114 males and 36 females of age group between 37 and 72 years (56.83 ± 11.36 years)) with no other disease were considered as control group.

### 2.2. Collection and Storage of Plasma

Venous blood was collected in EDTA tubes. Plasma was immediately separated from the cells by centrifugation at 3000 rpm for 10 min. Plasma samples for the measurement of prolidase activity and oxidative stress markers were stored at −80°C until being used. Each sample was thawed at room temperature for performing every test; repeated thaw was avoided.

### 2.3. Measurement of Plasma Prolidase Activity (PPA)

Diluting solution (1 mM MnCl_2_ in 6 mM Tris HCl buffer (pH 7.8–8.0)), standard proline solution (650 *μ*mol/L proline solutions in 0.45 mol/L trichloroacetic acid), and glycyl-L-proline solution (94 mmol/L Gly-l-Pro solution in 0.05 mol/L Tris HCl buffer containing 1 mmol/L of MnCl_2_ (pH 7.8–8.0)) were prepared. Chinard's reagent was prepared by mixing of 600 mL of glacial acetic acid with 400 mL of 6 mol/L orthophosphoric acid and 25 g of ninhydrin dissolved in the mixture at 70°C (in water bath). Before preparation of Chinard's reagent, 6 mol/L orthophosphoric acid was prepared by mixing of 407 mL of orthophosphoric acid (85%, *d* = 1.7) in 593 mL of distilled water.

*Procedure.* Plasma was diluted six times with diluting solution and preincubated for 24 hours at 37°C. For enzymatic reaction, experimental tube and control tube were selected. Experimental tube contained 100 *μ*L of 94 mmol/L Gly-l-Pro solution and 100 *μ*L of diluted and preincubated plasma, while control tube contained only 100 *μ*L of diluted and preincubated plasma. Both the tubes were incubated for 30 minutes at 37°C, and then the reaction was stopped by adding of 1 mL of 0.45 mol/L trichloroacetic acid. After the reaction is stopped, 100 *µ*L of diluted and non-incubated plasma was added in control tube. After this, supernatant was separated by centrifugation at 2000 rpm for 5 minutes. 0.5 mL of each tube supernatant was used for the measurement of enzyme activity.

Again four tubes were selected as blank tube, standard tube, experimental tube, and control tube for enzyme activity measurement. 1 mL of Chinard's reagent and 1 mL of glacial acetic acid were added to each tube. Then, 0.5 mL of above supernatant was added to respective experimental and control tube. Instead of supernatant, 0.5 mL of trichloroacetic acid (0.45 mol/L) and 0.5 mL of standard proline solution were added to the blank tube and standard tube, respectively. After this, all tubes were incubated at 90°C in water bath for 10 minutes for complex formation. Absorbance was measured at 515 nm. Spectrophotometer was adjusted to zero with the blank. PPA was measured according to following formula [14]:

(1) $$\begin{array}{c} \frac{E-C}{S}\times \left[S\right]\times 2.4=\text{mmol}\cdot \text{mi}{\text{n}}^{-1}\cdot {\text{L}}^{-1}. \end{array}$$

*E* is experimental tube absorbance, *C* control tube absorbance, *S* standard tube absorbance, and [*S*] concentration of the substrate in mmol/L (94 mmol/L). PPA was represented as mmol min^−1^ L^−1^.

### 2.4. Measurements of Total Antioxidant Status (TAS)

Blood plasma TAS was measured by the colorimetric method developed by Erel, 2004 [15]. 200 *μ*L of reagent-1 (*o*-dianisidine (10 mM), ferrous ion (45 *μ*M) in the Clark and Lubs solution (75 mM, pH 1.8)) was added to 5 *μ*L of plasma and then 10 *μ*L of reagent-2 (7.5 mM of H_2_O_2_ in the Clark and Lubs solution) was added. Absorbance was taken at 444 nm. Color formation was calibrated with Trolox and results were expressed in millimolar Trolox equivalent per liter (mmol Trolox Eq/L).

### 2.5. Measurements of Total Oxidant Status (TOS)

Blood plasma TOS was measured by the colorimetric method developed by Erel, 2005 [16]. 225 *μ*L of reagent-1 (150 *μ*M xylenol orange, 140 mM NaCl, and 1.35 M glycerol in 25 mM H_2_SO_4_ solution, pH 1.75) and 11 *μ*L of reagent-2 (5 mM ferrous ion and 10 mM* o*-dianisidine in 25 mM H_2_SO_4_ solution) were added to 35 *μ*L of plasma in sequence. Absorbance was taken as end point measurement, bichromatic at 560 nm (main wavelength) and 800 nm (secondary wavelength). The first absorbance was taken after mixing of reagent-1 and plasma (as blank) and last absorbance was taken after 4 minutes of mixing of reagent-2. The assay was calibrated with H_2_O_2_ and the results were expressed in micromolar H_2_O_2_ equivalent per liter (*μ*mol H_2_O_2_ Eq/L).

### 2.6. Measurements of Oxidative Stress Index (OSI)

OSI was calculated as ratio of TOS (*μ*mol H_2_O_2_ Eq/L) to TAS (mmol Trolox Eq/L) [11, 14].

### 2.7. Statistical Analysis

Standard statistical methods, Student's *t*-test, ANOVA, and Pearson's correlation were used. Nonparametric Mann-Whitney *U* test, Spearman's rho correlation, and Kruskal Wallis test were used if distributions were not normal. A *P* value less than 0.05 (*P* < 0.05) was considered significant. Data were representation as mean ± SD (standard deviation). All statistical calculations were done by use of SPSS 16.0 and Graph pad software. Interassay and intraassay precision performances of the assay were calculated for PPA, TAS, and TOS. Interassay and intraassay % of coefficient of variation for PPA, TAS, and TOS were 8.65% and 5.00%, 7.82% and 4.00%, and 6.51% and 4.68% observed, respectively.

## 3. Results

### 3.1. Plasma Prolidase Activity (PPA)

Our results showed that plasma prolidase activity in cases (40.30 ± 27.13 mmol min^−1^ L^−1^ with range 1.47 to 88.45 mmol min^−1^ L^−1^) was more significantly decreased than controls (61.70 ± 15.92 mmol min^−1^ L^−1^ with range 35.24 to 105.97 mmol min^−1^ L^−1^) (*P* < 0.001, Table 1).

### 3.2. Total Antioxidant Status (TAS)

TAS levels in cases (1.69 ± 0.47 mmol Trolox Eq/L, with range 0.95 to 2.76 mmol Trolox Eq/L) were more significantly decreased than healthy controls (1.93 ± 0.40 mmol Trolox Eq/L with range 1.18 to 2.56 mmol Trolox Eq/L) (*P* = 0.006, Table 1).

### 3.3. Total Oxidant Status (TOS) and Oxidative Stress Index (OSI)

TOS and OSI levels in cases (16.55 ± 3.28 *μ*mol H_2_O_2_ Eq/L and 11.02 ± 5.03 AU, resp.) were more significantly increased than controls (12.61 ± 2.57 *μ*mol H_2_O_2_ Eq/L and 7.13 ± 3.06 AU, resp.) (all *P* < 0.001, Table 1).

### 3.4. Correlative Observations

In cases, no correlations were observed between PPA and TOS and OSI (*r* = 0.202, *P* = 0.128 and *r* = 0.246, *P* = 0.063, resp.), while significant negative correlation was observed between PPA and TAS (*r* = −0.262, *P* = 0.047). In cases, strong negative and significant correlation were observed between TOS versus TAS and TAS versus OSI (*r* = −0.933, *P* < 0.001 and *r* = −0.912, *P* < 0.001, resp.), while significant, strong, and positive correlation was observed between TOS and OSI (*r* = 0.962, *P* < 0.001).

### 3.5. Altered Values of PPA, TAS, TOS, and OSI with Duration of PD

For the evaluation of PPA, TAS, TOS, and OSI with disease duration, total 240 cases were categorized as disease duration ≤2 years (140 subjects) and disease duration greater than 2 years (100 subjects, Table 2). PPA in cases with disease duration greater than 2 years (38.44 ± 25.43 mmol min^−1^ L^−1^) were nonsignificantly decreased compared to disease duration ≤2 years (43.13 ± 29.89 mmol min^−1^ L^−1^) (*P* = 0.747), while TAS was significantly decreased (*P* < 0.001). TOS and OSI in cases with disease duration greater than 2 years were more significantly increased than disease duration ≤2 years (*P* < 0.001) (Table 2).

On the basis of age, patients with PD and controls were divided into age group less than 50 years (Gp1: 32 subjects as cases and 24 subjects as controls), age group 50 to 59 years (Gp2: 124 subjects for cases and 75 subjects for controls), and age group more than 60 years (Gp3: 84 subjects for cases and 51 subjects for controls, Table 3). Observed PPA, TOS, TAS, and OSI values in cases and controls for age groups <50 years, 50–59 years, and >60 years were tabulated in Table 3.

In the cases, the differences of values of PPA, TOS, TAS, and OSI between male and female gender were nonsignificantly altered (data not shown). On the epidemiological analysis of the gender for the case group, out of total cases (*n* = 240, mean age 56.39 ± 8.92 years), it was observed that the number of males (*n* = 180, 75%, and mean age 54.73 ± 9.01 years) was three times greater than females (*n* = 60, 25%, and mean age 61.37 ± 6.71 years). This data was divided into different age ranges, 35–39 (6, male; nil, female), 40–49 (26, male; 6, female), 50–59 (67, male; 18, female), and 60–70 years (81, male; 36, female).

## 4. Discussion

Proline deficiency in human leads to mental retardation and it is expected that this proline deficiency may be due to prolidase deficiency [17]. In our present study, it was observed that plasma prolidase activity was more decreased in patients with PD than healthy subjects. Within PD patients, range of PPA was 1.47–88.45 mmol min^−1^ L^−1^, while in healthy volunteers the range of PPA was 35.24–105.97 mmol min^−1^ L^−1^ (Table 1).

Proline rich protein plays as key mediator of apoptosis and/or provides signal for induction of apoptosis [18], which leads to neuronal cells loss [19]. In present study we observed that plasma prolidase activity decreases with increase in duration of PD and may result in increase in concentration of proline rich dipeptides. It may lead to enhanced apoptotic signal and neuronal cell loss.

Polypeptide containing proline rich region plays a key role in aggresomes formation, while lack in proline rich region cannot allow the formation of aggresomes [20]. It has been reported that aggresomes formation leads to neuronal death in patients with PD [21, 22]. As our study suggests in the case of PD plasma prolidase activity decreases; thus it can increase the chance of accumulation of glycyl-l-proline peptides and other polypeptides containing c-terminal glycyl-l-proline, which may lead to aggresomes formation and neuronal death in patients with PD.

Most of neurofilaments (NF) proteins phosphorylation occurs in the carboxy-terminal tail domain of proline directed Ser/Thr residue [23]. NF of normal neurons has been predominantly phosphorylated in axonal region, while in the case of PD, NF proteins have been aberrantly hyperphosphorylated in the region of cell bodies [24]. It is seen that blockage of axonal transport in the cell body of neuron and neuronal cell death were observed with increase in NF phosphorylation [23]. Thus, it seems that proline in peptides is directly or indirectly involved in increasing signaling cascade for phosphorylation of NF proteins and finally leads to blockage of axonal transport and neuronal cell death. In our present study, we have observed decreased plasma prolidase activity in patients with PD; thus the possibility of peptides containing c-terminal proline in body increases, which may be involved in increased phosphorylation and neuronal cell death.

Altered oxidative stress, mitochondrial dysfunction, inflammation, and environmental factor were reported in patients with PD [25, 26]. In normal situation of cellular system, oxidant and antioxidant production are balanced by different cellular reactions [27, 28]. The rates of production and destruction of oxidants and antioxidants are in balance state, referred to as oxidative balance. When levels of oxidants or antioxidants or both are altered, the situations of oxidative stress are developed [29]. Different research groups have described the altered levels of oxidants and antioxidants in the term of altered value of lipid peroxidation, reactive oxygen species, superoxide dismutase, glutathione, and reactive nitrogen species in the case of patients with PD [25–27, 30]. Recently, Kirbas et al., 2014, have reported a study on paraoxonase, TAS, and TOS level in the patients with PD [13]. In our present study, we observed that status of PPA was nonsignificantly correlated to TOS and OSI in the patients with PD. Patients with PD have increased plasma TOS and OSI compared to healthy individuals, while PPA and TAS levels in patients with PD were significantly decreased as compared to normal healthy individuals (*P* < 0.01, Table 1).

PPA, TOS, TAS, and OSI are considered as oxidative stress markers for biological system. In general, prolonged increased oxidant level above the normal threshold leads to exhaustion and therefore decrease in antioxidant status in human. This leads to change in OSI value. In the case of patients with PD, we observed that decreased PPA was positively, linearly correlated to increase in TOS (*P* = 0.128) and OSI (*P* = 0.063), but all these were nonsignificant, while in patients with PD, decreased TAS were negatively, linearly correlated to PPA which was significant (*P* = 0.047). Different investigators have reported that increased oxidants were related to decreased antioxidants in patients with PD [10, 22–24, 27]. On the same patterns, in patients with PD, we observed that increased TOS were linearly correlated to increased OSI (*P* < 0.001), while strong negative, linear correlations were observed between TOS and TAS (*P* < 0.001). It should be noted that positive linear correlation between decreased PPA and increased TOS in patients with PD will need to be explored further for better clarification.

It is well accepted that increased oxidants and decreased antioxidants are associated with increase in age and lead to loss of neuron in brain and substantia nigra [25–28]. Hattiangady et al., 2010, have been reported that loss of neurons and cognitive functions was related to age [31]. Different investigators reported that neuronal cells loss was associated with increase in duration of Parkinson's disease [32]. In our present study, we observed that PPA and TAS in patients with PD were decreased with increase in duration of disease, while TOS and OSI were increased with increase in duration of disease (Table 2). Thus it seems that decreased PPA and TAS and increased TOS and OSI were associated with progression of disease and might be responsible for neuronal cell loss in patients with PD.

Results of oxidative stress markers were also analyzed in different age groups (Gp1, Gp2, and Gp3, Table 3) in patients with PD and healthy volunteers. We observed that age group >60 years (Gp3) has decreased PPA compared to age group between 50 and 59 years (Gp2) in patients with PD, indicating that higher age groups have decreased PPA status compared to lower age groups in patients with PD. On analysis of Table 3, it is concluded that higher age groups have increased TOS and OSI and decreased TAS compared to lower age groups in patients with PD, but all are nonsignificant.

It is well accepted that higher age and male sex are more prone to PD. In American population, the onset of PD rarely occurs before the age of 40 years and the incidence of disease rapidly increased with rise in age (after 60 years) [33]. In our studied case group, all the patients with PD were selected from the two state of North India (Uttar Pradesh and Bihar), who attended the OPD of Sir Sunder Lal hospital, Institute of Medical Sciences, BHU, Varanasi, during the period of September, 2011, to August, 2014. In this study, 3 : 1 male and female ratio for Parkinson's disease was observed. It was also observed that early onset of PD for male was seen in age range of 35–39 years, while for female it was 40–49 years. Below 40 years' onset of PD for female was not observed. For PD, the mean age of male was 54.73 ± 9.01 years, while for female it was 61.37 ± 6.71 years. Thus, males are more prone to PD in reference to gender, as well as at lower age, than female.

## 5. Conclusions

Results of our study concluded that plasma prolidase activity and total antioxidant status were significantly decreased while total oxidant status and oxidative stress index were significantly increased in the patients with PD as compared to healthy volunteers. With the increase in disease duration (progression of disease) PPA and TAS were decreased, while TOS and OSI were increased. With age, similar results are seen. Thus, the decreased status of PPA and increased oxidative stress were associated with progression of disease and higher age group and may be responsible for the pathogenesis of Parkinson's disease. On the epidemiological analysis of PD cases, it is concluded that males are more prone to early onset of PD.

## Figures and Tables

**Table 1:** Representation of plasma prolidase activity, total oxidant status, total antioxidant status, and oxidative stress index in cases and controls.

Conditions/parameters	Plasma prolidase activity (mmol min^−1^ L^−1^)	Total oxidant status (*μ*mol H_2_O_2_ Eq/L)	Total antioxidant status (mmol Trolox Eq/L)	Oxidative stress index (arbitrary unit)
Cases (*n* = 240)	40.30 ± 27.13 (*R* = 1.47 to 88.45, median = 39.13)	16.55 ± 3.28 (*R* = 9.80 to 28.91)	1.69 ± 0.47 (*R* = 0.95 to 2.76)	11.02 ± 5.03 (*R* = 3.55 to 30.43)

Controls (*n* = 150)	61.70 ± 15.92 (*R* = 35.24 to 105.97, median = 58.86)	12.61 ± 2.57 (*R* = 8.75 to 17.11)	1.93 ± 0.40 (*R* = 1.18 to 2.56)	7.13 ± 3.06 (*R* = 3.37 to 14.50)

*P* value (two-tailed)	<0.001^**^	<0.001^*^	0.006^*^	<0.001^*^

Significant (Sig.) *P* value less than 0.05 was considered. Data are mean ± standard deviation, *n* = number, *R* = range, ∗ = value according to Student's *t*-test, and ∗∗ = value according to Mann-Whitney *U* test.

**Table 2:** Representation of PPA, TOS, TAS, and OSI with duration of disease in patients with PD.

Duration of Parkinson's disease	*N* (240)	PPA (mmol min^−1^ L^−1^)	TOS (*μ*mol H_2_O_2_ Eq/L)	TAS (mmol Trolox Eq/L)	OSI (arbitrary unit)
≤2 years (*R* = 0.5 to 2.0 years)	140	43.13 ± 29.89	14.71 ± 2.39	1.98 ± 0.38	7.92 ± 2.53

>2 years (*R* = 2.5 to 14.0 years)	100	38.44 ± 25.43	19.34 ± 2.33	1.27 ± 0.16	15.73 ± 4.14

*P* value		0.747^**^	<0.001^*^	<0.001^*^	<0.001^*^

Significant (Sig.) *P* value less than 0.05 was considered. Data are mean ± standard deviation, *R* = range, ∗ = value according to Student's *t*-test, and ∗∗ = value according to Mann-Whitney *U* test.

**Table 3:** Representation of PPA, TOS, TAS, and OSI in different age groups of cases and controls.

Parameters	Cases (*n* = 240)			Sig. value	Controls (*n* = 150)			Sig. value
	<50 years (Gp1) (*n* = 38)	50–59 years (Gp2) (*n* = 85)	≥60 years (Gp3) (*n* = 117)		<50 years (Gp1) (*n* = 24)	50–59 years (Gp2) (*n* = 51)	≥60 years (Gp3) (*n* = 75)	
PPA mmol min^−1^ L^−1^	28.06 ± 34.29	42.91 ± 25.54	37.12 ± 29.86	0.581^**^	61.71 ± 14.68	55.51 ± 13.57	63.87 ± 18.55	0.527^**^

TOS *μ*mol H_2_O_2_ Eq/L	13.30 ± 3.02	16.31 ± 2.79	17.26 ± 3.91	0.210^*^	12.57 ± 2.60	11.54 ± 2.82	13.04 ± 2.48	0.478^*^

TAS mmol Trolox Eq/L	2.28 ± 0.21	1.72 ± 0.46	1.60 ± 0.47	0.130^*^	1.93 ± 0.39	2.01 ± 0.43	1.89 ± 0.41	0.837^*^

OSI Arbitrary unit	5.91 ± 1.87	10.47 ± 3.92	12.40 ± 6.38	0.182^**^	7.05 ± 2.90	6.27 ± 3.18	7.54 ± 3.35	0.643^**^

Gp = group, *n* = numbers of subject, ∗ = values according to one-way ANOVA, and ∗∗ = values according to Kruskal Wallis test.
